# Miscellany

**Published:** 1893-04

**Authors:** 


### Miscellany.

An open competitive examination of candidates for the position of Female Physician in the State Hospital service, will be held at the rooms of the State Civil Service Commission, in the Capitol, Albany, on Wednesday, April 12, 1893, at ten o'clock A. M.

Applicants must be residents of the State of New York during the year last past, and must be graduates of a legally incorporated medical college, and must have had one year's experience in a hospital, or three years' experience in the general practice of medicine. Salary, $1,200 per annum and board.

For application blanks, address Clarence B. Angle, Secretary New York Civil Service Commission, Albany, N. Y.

Albany, N. Y., March 10, 1893. THOMAS CARMODY,
*Chief Examiner.*

---

### NEW BY-LAWS, PAN-AMERICAN MEDICAL CONGRESS.

*Languages:* By-Law IX.—Papers may be read in any language, providing that authors of the same shall furnish the Secretary-General with an abstract not exceeding 600 words in length in either of the official languages, (English, Spanish, French, or Portuguese,) by not later than July 10, 1893, and, providing further, that a copy of each such paper shall be furnished in either of the official languages, at or before the time of the meeting to the secretary of the section before which the same shall be read. Remarks upon papers may be made in any language, providing that members making such remarks shall furnish a copy of the same in either of the official languages before the adjournment of the session.

*Publications:* By-Law X.—All papers read either in full or by part shall be immediately submitted for publication in the transactions, (Special Regulation, 3,) but authors may retain copies and publish the same at their pleasure after the adjournment of the Congress.

*Constituent Organizations:* By-Law XI.—All medical, dental, and pharmaceutical organizations, the titles of which have been transmitted with approval to the Committee on Organization, or which may hereafter be transmitted with approval to the Executive Committee by any member of the International Executive Committee, each for his own county, shall be subject to election by the Executive Committee, approved by the President as constituent bodies of the First Pan-American Medical Congress, and each organization thus constituted shall have the right to designate as delegates all of its members attending the Congress, but no such organization shall meet at the time and place of meeting of the Congress as a distinct body, providing that the secretary of each such constituent body shall furnish a list of officers and a statement of the number of members of his respective organization to the Secretary-General not later than sixty days before the meeting of the Congress, and shall forward a list of delegates chosen to reach the Secretary-General before the opening of the Congress.

February 22, 1893. *By the Executive Committee.*

---

### PAN-AMERICAN CONGRESS BULLETIN.

*Section on Medical Pedagogics.*—The pedagogic section will devote its attention especially to the history of the development of medical education in America. In the papers presented by leading teachers, recent advances in methods of instruction will be considered.

The *art of teaching*, which is regarded as a study of great interest in other branches of learning, has received hitherto but little attention from the medical profession.

The Section in Medical Pedagogics will, therefore, be made a prominent feature of the Congress, and it is hoped that those interested in medical education will coöperate in the work of this section by being present and by actively engaging in the discussion of subjects presented.

Any inquiries or communications may be made through the secretaries undersigned.

J. COLLINS WARREN, M. D.,
*Executive President, Boston, Mass.*
CHARLES L. SCUDDER, M. D.,
*English-Speaking Sec'y, Boston, Mass.*
WM. F. HUTCHINSON, M. D.,
*Spanish-Speaking Sec'y, Providence, R. I.*

To Make Steel Instruments as Bright as New.—(*Medical Brief.*)—Clean the instruments by rubbing with wood ashes and soft water. Then soak them in a weak solution of hydrochloric acid in water (about ten to fifteen drops to the fluid ounce) for a few hours, to remove the remaining rust and grease. Then wash them well in pure soft water. The next step is to place them in a bath consisting of a *saturated* solution of tin chloride. Let them remain ten to twenty-four hours, according to the coating desired. When removed from the bath, wash them clean in pure water and dry well. When the job is well done, the steel will appear as if nickle plated.—*Ex.*

---

Concerning Disinfection of Banknotes.—According to the *Journal Russkaho Obshtchestva Okhranenia Narodnaho Zdravia* (Journal of the Russian Society for the Protection of National Health), No. 10, 1892, p. 756, the Roumanian Government has recently issued an order to the effect that all banknotes sent to the country from cholera-stricken localities are to be subjected to disinfection with a carbolic solution. The practice has shown that Russian, German, French, and Servian banknotes do not undergo any discoloration even when treated by a ten per cent. solution of carbolic acid. Meanwhile, the Austrian and Italian lose their color, and ultimately transform into worthless bits of white paper, even when they come in contact with a six per cent. solution of the drug.—*St. Louis Medical and Surgical Journal.*

---

The Bank-note Bacillus—This is the name of a new variety of microbe, thriving, according to a British bacteriologist, on notes of the Bank of England. He believes that a deadly and sure medium for the migration of bacteria is the bank-note of small denomination. Some foreign notes were "experimented" on, and in two cases 19,000 microbes were discovered vegetating on a single note. Among them were identified microbes of tuberculosis, diphtheria, and scarlatina. The great majority of the microbes belonged to a peculiar type which, it is suggested, should be called the Bank-note Bacillus.—*Medical Record, Jan. 21, 1893.*

